# Comparative efficacy of non-pharmacological interventions for Parkinson’s disease with constipation: a systematic review and network meta-analysis

**DOI:** 10.3389/fneur.2025.1579556

**Published:** 2025-06-02

**Authors:** Peiying Zhang, Xiaojuan Su, Xuan Han, Dingmeng Zhao, Jinyan Wang, Yanyi Yang, Hejiang Ye

**Affiliations:** ^1^College of Clinical Medicine, Chengdu University of Traditional Chinese Medicine, Chengdu, China; ^2^Hubei Provincial Hospital of Traditional Chinese Medicine, Wuhan, China; ^3^Institute of Information Engineering, Chinese Academy of Sciences, Beijing, China; ^4^Hospital of Chengdu University of Traditional Chinese Medicine, Chengdu, China

**Keywords:** Parkinson’s disease, constipation, non-pharmacological interventions, network meta-analysis, therapeutic effect

## Abstract

**Background:**

This network meta-analysis aims to evaluate the comparative efficacy of non-pharmacological interventions on Parkinson’s disease (PD) with constipation.

**Methods:**

A comprehensive search was conducted in seven major databases (CINAHL, the Cochrane Central Register of Controlled Trials [CENTRAL], Embase, PubMed, Web of Science, Chinese National Knowledge Infrastructure [CNKI], and Wanfang) up to August 2024. Eligible randomized controlled trials (RCTs) that evaluated non-pharmacological interventions for PD with constipation were included. Methodological quality was assessed using the Cochrane Risk of Bias tool, and a frequentist network meta-analysis (NMA) was performed using STATA 18 to estimate relative treatment effects.

**Results:**

From 2084 initially identified records, 12 RCTs (*n* = 881 patients) met inclusion criteria. The four interventions evaluated included complementary and alternative medicine (CAM), evidence-based nursing (EBN), physical agents (PAs), and traditional Chinese medicine (TCM). Direct comparisons revealed significantly superior efficacy for both EBN and PAs compared to control conditions (*p* < 0.05). The NMA demonstrated consistent superiority of PAs and EBN over passive control, placebo, and sham interventions (all *p* < 0.05), with the following efficacy hierarchy: PAs (most effective) > EBN > CAM > TCM (least effective).

**Conclusion:**

Our findings suggest that non-pharmacological approaches, particularly PA-based interventions, may offer clinically meaningful benefits for constipation management in PD. Nevertheless, the relatively small number of available studies and methodological limitations in several trials necessitate cautious interpretation. Further rigorously designed RCTs are warranted to confirm these preliminary observations and establish optimal treatment protocols.

**Systematic review registration:**

https://www.crd.york.ac.uk/PROSPERO/, CRD42024565248.

## 1 Introduction

Parkinson’s disease (PD) has emerged as a growing global health challenge, with its prevalence reaching alarming levels—approximately 6.1 million cases worldwide in 2016, representing a 2.4-fold increase since 1990 (1, 2). Epidemiological studies consistently demonstrate upward trends in PD incidence, prevalence, and associated disability burdens across all regions in recent decades (2–4). What often escapes clinical attention is that constipation, along with other non-motor symptoms such as rapid eye movement (REM) sleep behavior disorder and mood disturbances, frequently manifests years before the characteristic motor signs appear. Constipation, in particular, as a gastrointestinal dysfunction, not only diminishes patients’ quality of life but also creates substantial societal and economic burdens (5–8).

Effective and practical management of PD with constipation presents a pressing clinical challenge (5). While conventional drug treatments exist, their utility is frequently constrained by suboptimal efficacy and adverse reactions, leaving many patients inadequately treated (9–11). This therapeutic gap has accelerated interest in non-pharmacological alternatives targeting multiple pathophysiological mechanisms—from gut microbiome modulation to neuromuscular coordination improvement (12–19). Emerging evidence suggests that these approaches may offer safer and potentially more sustainable relief for bowel dysfunction in PD patients (5, 11, 20).

Despite promising therapeutic advances in this field, the comparative effectiveness of various non-drug interventions remains uncertain. Traditional meta-analyses have been constrained by their inability to simultaneously evaluate multiple treatment modalities. Network meta-analysis (NMA) represents a methodological breakthrough that overcomes this limitation by integrating both direct and indirect comparative evidence (21–23). Our study used this innovative approach to systematically assess and rank the efficacy of diverse non-pharmacological strategies for managing PD with constipation in randomized controlled trials (RCTs), aiming to provide much-needed clarity for clinical decision-making.

## 2 Methods

### 2.1 Protocol

Preferred Reporting Items for Systematic Reviews and Meta-Analysis (PRISMA) guidelines and the Cochrane Handbook for the Systematic Review of Interventions were selected to guide the normalization of this systematic review and NMA (24, 25). The study protocol was prospectively registered with PROSPERO (CRD42024565248).

### 2.2 Data sources and search strategy

To capture all relevant evidence, we conducted exhaustive searches across seven major biomedical databases: CINAHL, the Cochrane Central Register of Controlled Trials (CENTRAL), Embase, PubMed, Web of Science, Chinese National Knowledge Infrastructure (CNKI), and Wanfang. The search period spanned from their inception through August 2024. The search strategy used a combination of three keywords and their related synonyms, derived from Medical Subject Headings (MeSH), applying suitable truncation and Boolean operators in the title and abstract sections: “Parkinson’s disease,” “constipation,” and “clinical trial.” To account for variations in Chinese academic writing conventions, we omitted RCT-specific search terms when querying Chinese databases to ensure comprehensive literature coverage. The complete search strategies are available in Supplementary material 2. Additionally, the reference lists from eligible articles and relevant systematic reviews were manually screened for additional research.

### 2.3 Inclusion criteria

Population, Intervention, Comparison, Outcome, and Study Design (PICOS), as a search strategy tool for structuring clinical research questions in connection with evidence syntheses, was used to guide the definition of experiments selected for inclusion (24).

#### 2.3.1 Population

The study population comprised individuals presenting with both resting tremor and defecation difficulties, and needed to meet either: (1) the diagnostic criteria of the UK Parkinson’s Disease Society Brain Bank, or (2) the 2015 diagnostic criteria for PD revised by the Movement Disorder Society (26). Additionally, all enrolled subjects were required to satisfy the Rome III or IV diagnostic thresholds for functional constipation (27). Patients at any stage of PD were included.

#### 2.3.2 Interventions

Any non-pharmacological treatments (e.g., acupuncture, catgut embedding, complementary, and alternative medicine) were eligible for inclusion regardless of frequency, duration, or intensity.

#### 2.3.3 Comparisons

The comparators had at least one inactive (placebo, sham treatment, waiting list, or usual care) or eligible active interventions.

#### 2.3.4 Outcomes

Eligible studies needed to provide a detailed evaluation of PD-related or constipation-specific symptoms using at least one of the three validated measurement tools: (1) Patient Assessment of Constipation Quality of Life (PAC-QoL) questionnaire, (2) 39-item Parkinson’s Disease Questionnaire (PDQ-39), or (3) Unified Parkinson’s Disease Rating Scale-III (UPDRS-III) (28–31).

#### 2.3.5 Study design

Only RCTs were included in the analysis.

### 2.4 Exclusion criteria

Studies meeting any of the following criteria were excluded: (1) participants did not meet the diagnoses of both PD and constipation; (2) experimental group inclusion of pharmacological treatments (regardless of administration route); (3) use of combined therapeutic approaches (e.g., acupuncture combined with enemas); (4) non-RCTs including meta-analyses and conference abstracts; or (5) insufficient reported data that remained unavailable after contacting corresponding authors.

### 2.5 Data selection and extraction

All identified studies were imported into EndNote 21 for duplicate removal. Two investigators (Zhang and Su) independently conducted the screening process in three phases: title review, abstract evaluation, and full-text assessment. Thereafter, quality assessment and data extraction were carried out. To ensure consistency, any disagreements were resolved by discussion or with a third-party adjudicator (Han). Data extracted included: first author, publication year, country, sample size, age, gender, disease duration, type of intervention and its duration, and outcome indicators. If a study had more than one follow-up time points, the assessment closest to the end of the intervention period was selected.

### 2.6 Risk of bias (RoB) within individual studies

The Cochrane Collaboration Risk-of-Bias (RoB) 2.0 tool was used to rigorously appraise the methodological quality of eligible studies by two independent reviewers (Zhang and Su) (32). The overall risk of bias was scored as low, some concerns, or high.

### 2.7 Certainty of evidence

We rated the certainty of evidence for primary outcomes using the Grading of Recommendations Assessment, Development, and Evaluation (GRADE) framework and GRADEpro website^1^ (33, 34). Based on GRADE’s five criteria for downgrading evidence (risk of bias, inconsistency, indirectness, imprecision, and publication bias), we classified the certainty of evidence into four levels: high, moderate, low, or very low.

### 2.8 Statistical analyses

Mean differences (MDs) and standard deviations (SDs) were used as effective indicators for continuous variables. When data were reported as medians and interquartile ranges and showed no marked deviation from normality, they were optimally estimated as MDs and SDs using sample size, median, and quartiles (35–37). All included trials reported pre- and post-intervention measurements, enabling computation of change scores according to Cochrane Handbook methodology (38). To facilitate comparison across studies using different measurement scales, we expressed effect sizes as standardized mean differences (SMDs) with 95% confidence intervals (CIs).

All analyses were performed using STATA 18 with the statistical package (21, 39). Heterogeneity was assessed through Cochrane Q-statistics and the I^2^ statistic. We used the fixed-effects model when heterogeneity was negligible (I^2^ ≤ 50%, *p* ≥ 0.1), switching to a random-effects model for significant heterogeneity (I^2^ > 50%, *p* < 0.1) (32).

A random-effects NMA was constructed within a frequentist framework using STATA 18 (40). A network plot chart was used to reflect the classification of interventions and the correlation between interventions, as node sizes reflected sample sizes and connecting line thickness indicated the number of comparative studies. The coherence assumption was evaluated based on study characteristics. We evaluated consistency between direct and indirect evidence using both local (node-splitting) and global (inconsistency model) methods when closed loops existed in the evidence network. For networks without loops, we defaulted to consistency models (41).

Relative rankings of the competing interventions were estimated using surface under the cumulative ranking curve (SUCRA) probabilities, with higher values indicating better performance.

Sensitivity analyses were performed to evaluate the robustness of the NMA combination findings. To explore the observed heterogeneity using prespecified covariates, subgroup analyses and meta-regression analyses were performed for baseline characteristics (e.g., region, sex, and sample size) and trial characteristics (e.g., interventions, follow-up time, and outcome measures) of included studies. When subgroup stratification failed to explain heterogeneity, we sequentially excluded individual trials to identify influential outliers. The risk of publication bias for networks with 10 or more studies was assessed using the funnel plot and Egger’s test. Where significant asymmetry was detected, we applied the trim-and-fill method to estimate and adjust for potential missing studies (32, 42, 43).

## 3 Results

### 3.1 Study selection

**Figure 1:**
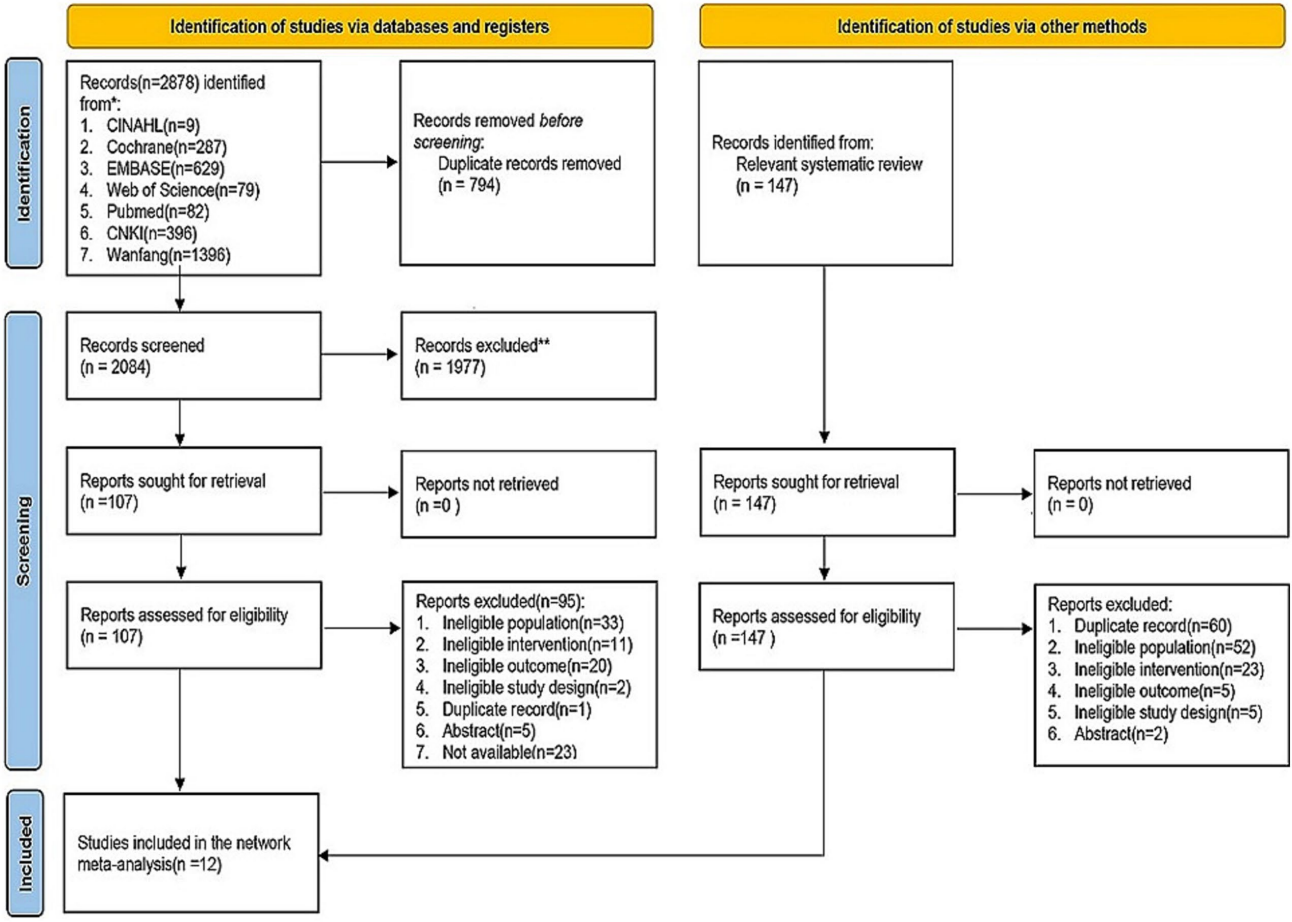
PRISMA flow diagram of study selection.

Following duplicate removal, a total of 2084 records were initially screened, and 1977 were excluded after title/abstract review. Full texts of the remaining 107 articles were scrutinized, and 95 of them failed to meet the inclusion criteria. Manual reference checks of included articles and relevant reviews yielded 147 additional records, though none qualified for final inclusion. Ultimately, 12 studies were retained for analysis (17–19, 44–52). A flow diagram of the study selection process is provided in Figure 1.

### 3.2 Characteristics of included trials

A total of 881 participants (experimental: 439, control: 442) from the 12 selected RCTs were included. Participants’ mean age ranged from 59.21 ± 19.51 to 76.77 ± 11.50 years, with 55.9% being male. Disease duration varied substantially across studies (3.14 ± 1.64 to 9.3 ± 6.7 years). Geographically, the studies originated from Iran (*n* = 1), Malaysia (*n* = 1), and China (*n* = 10), published between 2017 and 2023. Meanwhile, among the 12 baseline studies on demographic characteristics, 3 studies contained incomplete PD duration data (with missing, incorrect, ambiguous, and biased normality) (49, 51, 52), while 1 (51) lacked exact baseline age information. Complete study characteristics are shown in Table 1.

**Table 1:** General characteristics of included studies.

Author/Year	Region	Intervention	Mean follow-up	Sample		Male		Mean age		Mean duration	
				CON	EXP	CON	EXP	CON	EXP	CON	EXP
Li et al. (17)	China	PC vs. TCM	28 days	46	47	34	37	70.73 ± 8.88	69.82 ± 8.42	4.26 ± 1.18	4.24 ± 1.27
Li et al. (18)	China	PC vs. TCM	30 days	30	30	14	17	75.77 ± 11.30	76.77 ± 11.50	4.67 ± 1.77	4.60 ± 1.54
Song et al. (19)	China	SS vs. TCM	12 weeks	40	40	22	23	71 ± 6	70 ± 6	3.98 ± 2.56	3.14 ± 1.64
Zhan and Wang (44)	China	PC vs. TCM	48 days	20	20	11	8	63.14 ± 7.28	61.87 ± 6.25	5.75 ± 3.17	5.89 ± 4.62
Wang et al. (45)	China	PC vs. EBN	14 days	30	30	15	15	59.38 ± 18.48	59.21 ± 19.51	5.14 ± 2.51	5.26 ± 2.72
Yang et al. (46)	China	PL vs. CAM	12 weeks	63	65	42	31	69.64 ± 6.41	67.22 ± 6.46	6.51 ± 4.92	6.29 ± 4.47
Li et al. (47)	China	SS vs. TCM	4 weeks	39	39	18	17	63.74 ± 9.24	63.90 ± 7.34	6.05 ± 4.37	5.74 ± 3.95
Ghalandari et al. (48)	Iran	PL vs. CAM	8 weeks	13	14	7	8	68.54 ± 6.92	68.07 ± 6.68	6.00 ± 3.63	4.43 ± 2.38
Li et al. (49)	China	PC vs. TCM	12 weeks	83	83	43	45	66.9 ± 7.1	67.3 ± 8.1	N/A	N/A
Huang et al. (50)	China	SS vs. PA	4 weeks	24	24	12	14	69.6 ± 8.2	66.7 ± 9.6	9.3 ± 6.7	8.8 ± 5.9
Ibrahim et al. (51)	Malaysia	PL vs. CAM	8 weeks	28	27	17	16	N/A	N/A	N/A	N/A
Du et al. (52)	China	PC vs. CAM	12 weeks	23	23	10	16	66.65 ± 8.66	68.39 ± 7.55	N/A	N/A

CON, control; EXP, experimental; PC, passive control; TCM, traditional Chinese medicine; SS, Sham stimulation; EBN, evidence-based nursing; PL, placebo; CAM, complementary and alternative medicine; PAs, physical agents; N/A, not available, data missing, incorrect, ambiguous, or biased normality.

### 3.3 Type of intervention

In total, there were four different non-pharmacological interventions, summarized into four categories, including evidence-based nursing (EBN; *n* = 1), physical agents (PAs; *n* = 1), complementary and alternative medicine (CAM; *n* = 4), traditional Chinese medicine (TCM; *n* = 6). Detailed intervention definitions are shown in Table 2.

**Table 2:** Definitions of each intervention and control.

Intervention	Definition
Complementary and alternative medicine (CAM)	A variety range of medical practices and products that are not part of conventional medicine, such as nutritional supplements, dietary therapies, yoga, and meditation.
Evidence-based nursing (EBN)	The explicit and judicious use of theoretically derived, research-based information to make decisions about care to be delivered to individuals and to consider of individual needs.
Passive control (PC)	No treatment, waiting list control, treatment as usual, maintenance of daily activities, or standard of care from general practitioners.
Physical agents (PAs)	Physical therapy is applied externally to the point of pain (limb or trunk) without destroying or puncturing the skin, such as infrared radiation, repetitive transcranial magnetic stimulation, and magnetic stimulation.
Placebo (PL)	A drug or treatment with a similar appearance or methodology but no direct therapeutic effect, to eliminate the potential influence of the patient’s psychological and emotional state on the trial results.
Sham stimulation (SS)	A false or invalid stimulus is provided in an experiment for comparison with a true stimulus.
Traditional Chinese medicine (TCM)	A traditional medical system, based on the theory of Yin and Yang and the five elements, focuses on holistic regulation, syndrome differentiation and treatment, and treats diseases through herbs, acupuncture, massage and other methods.

### 3.4 Assessment of risk of Bias

**Figure 2:**
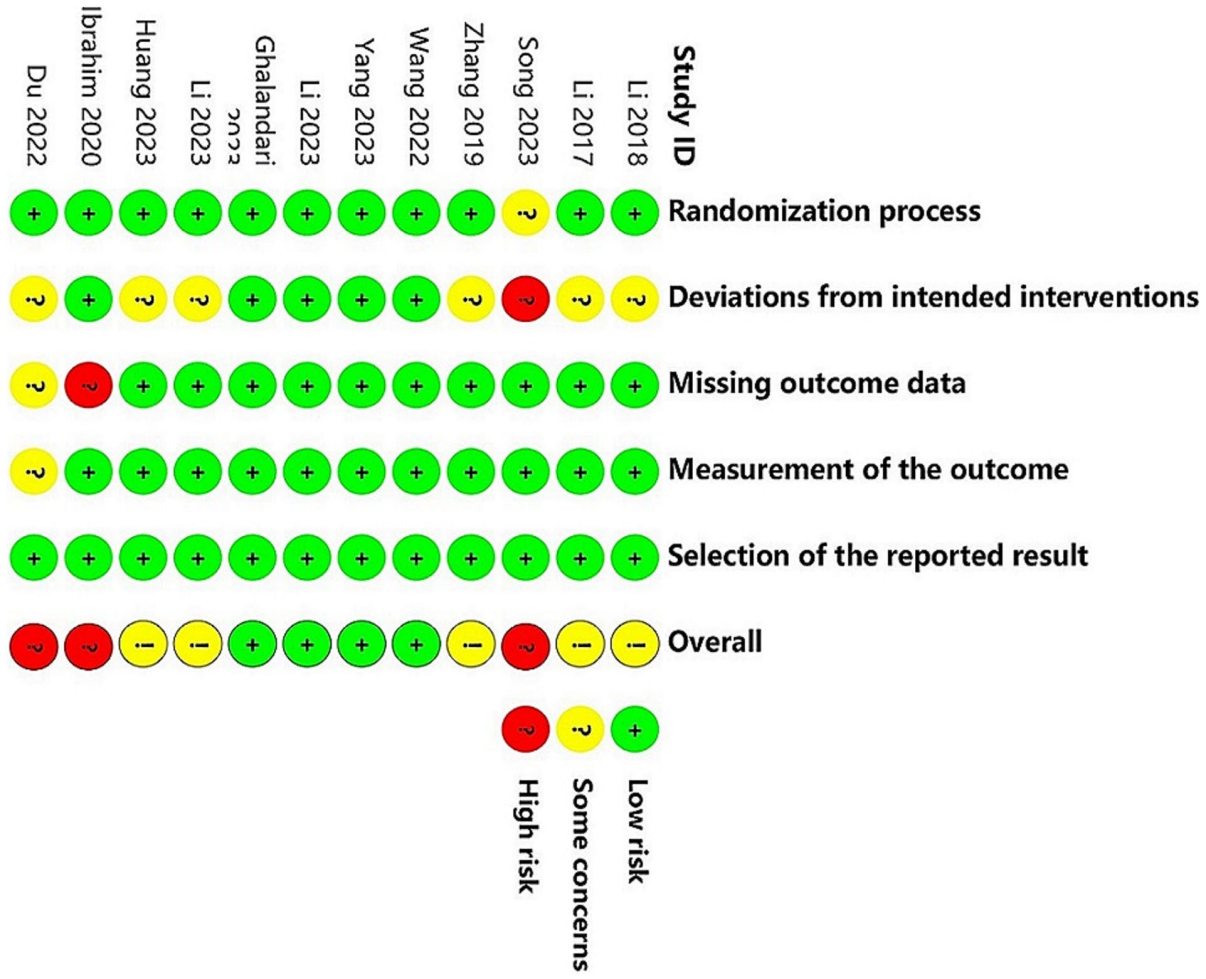
Assessment of risk of bias.

**Figure 3:**
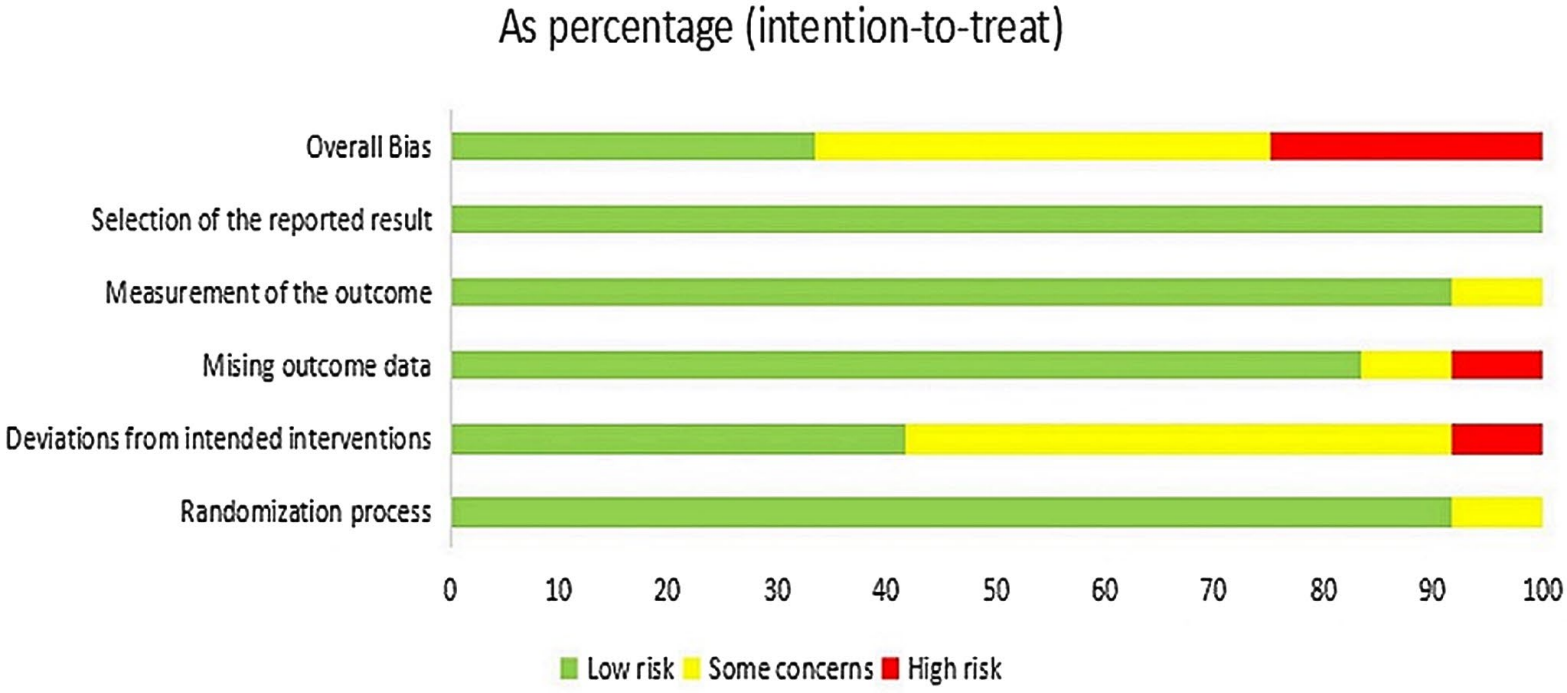
Assessment of risk of bias.

Figures 2, 3 present the assessment of the methodological quality across studies. Out of the 12 included studies, 4 (33.3%) were classified as having a low risk of bias, 5 (41.7%) raised some concerns, and 3 (25.0%) were deemed to have a high risk of bias. The crucial source of deviations is the lack of intervention blinding. Specifically, due to the nature of non-pharmacological treatments, it was challenging to blind both participants and providers.

### 3.5 Pairwise meta-analysis

The results of the conventional pairwise meta-analysis comparing non-pharmacological interventions with control treatment are presented in Supplementary material 3. Both EBN and PAs significantly demonstrated statistically superior effects in constipation management (*p* < 0.05).

### 3.6 Network meta-analyses for outcomes

**Figure 4:**
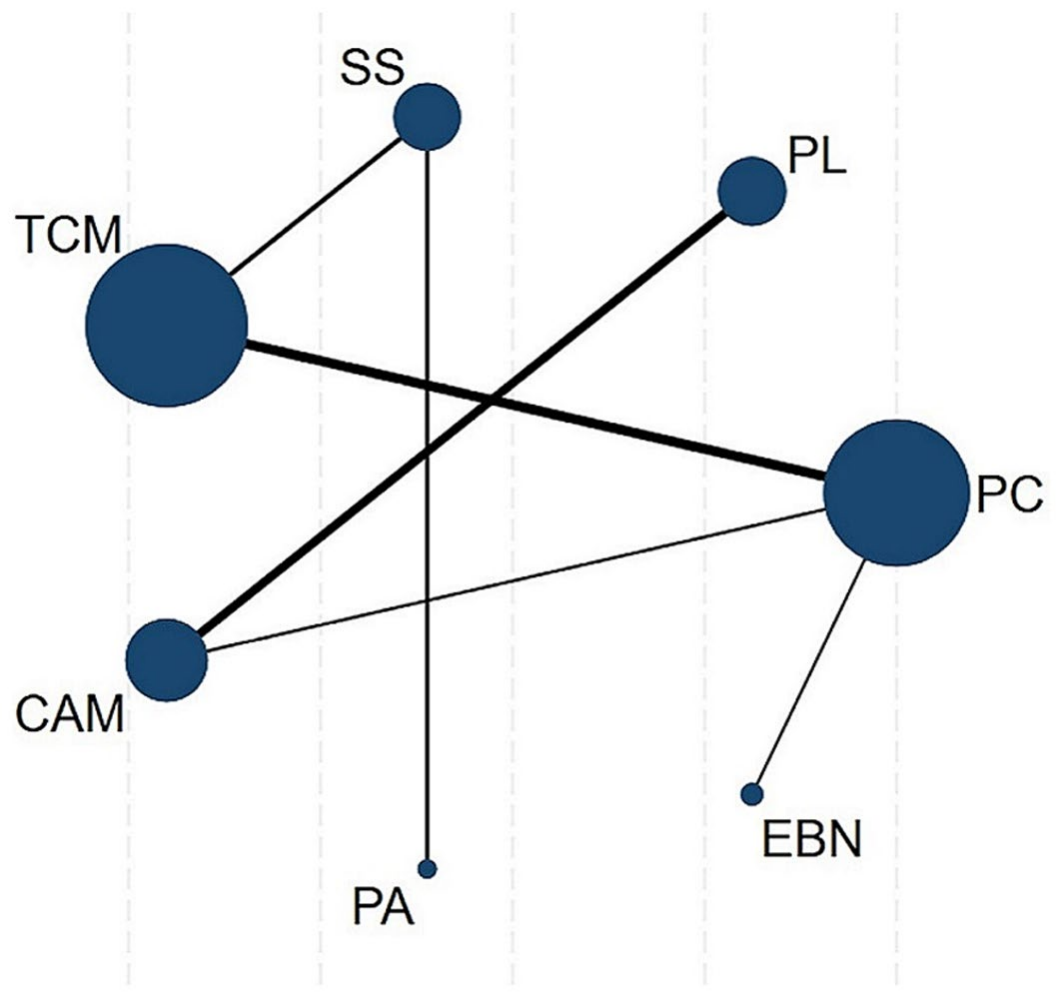
Network plot. Node sizes represent sample sizes, connecting line thickness indicates the number of comparative studies. CAM, Complementary and alternative medicine; EBN, evidence-based nursing; PAs, physical agents; PC, passive control; PL, placebo; SS, Sham stimulation; TCM, traditional Chinese medicine.

**Figure 5:**
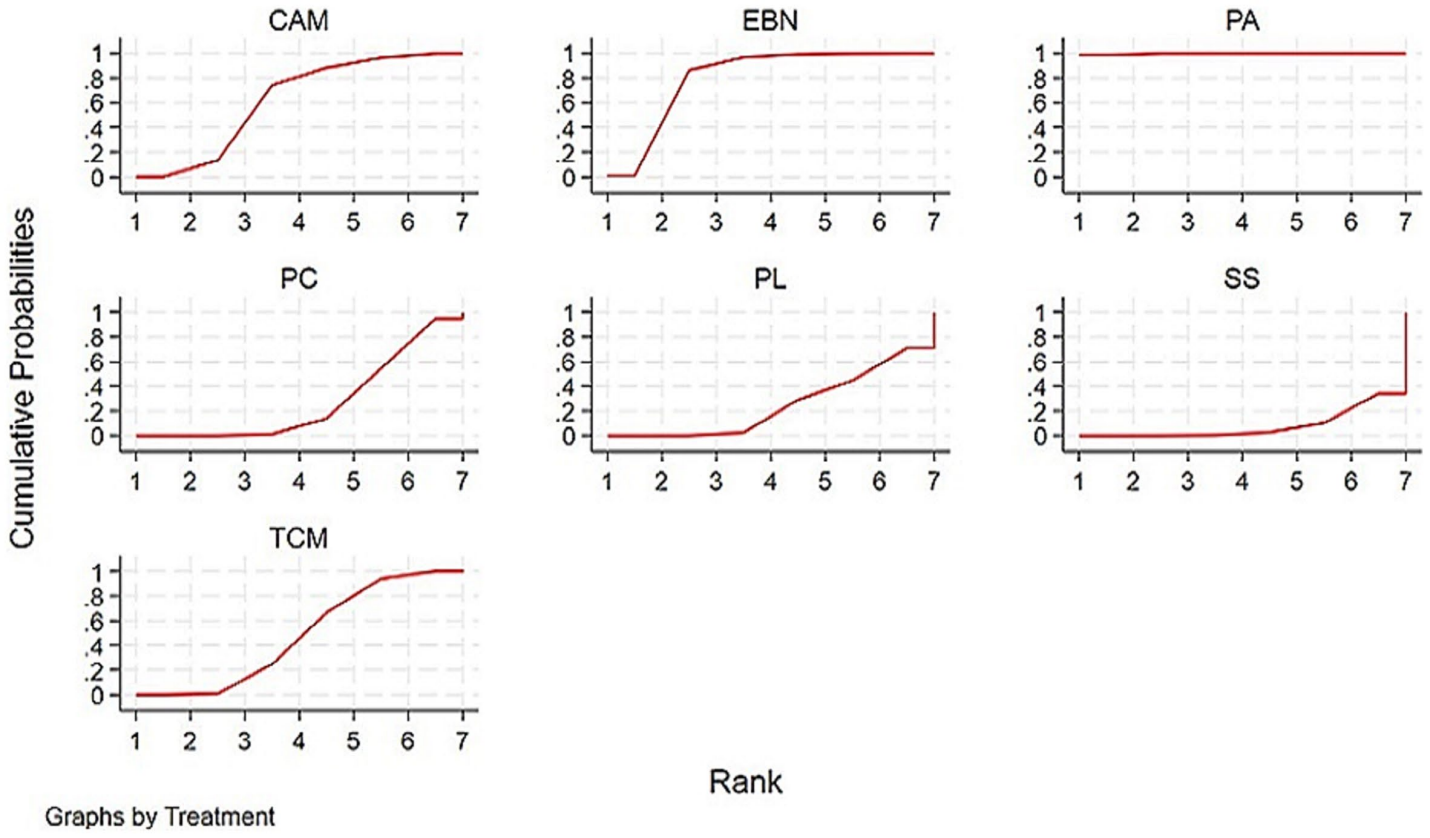
SUCRAs for PD with constipation. Interventions with larger cumulative ranking areas demonstrate superior efficacy. CAM, Complementary and alternative medicine; EBN, evidence-based nursing; PAs, physical agents; PC, passive control; PL, placebo; SS, Sham stimulation; TCM, traditional Chinese medicine.

**Figure 6:**
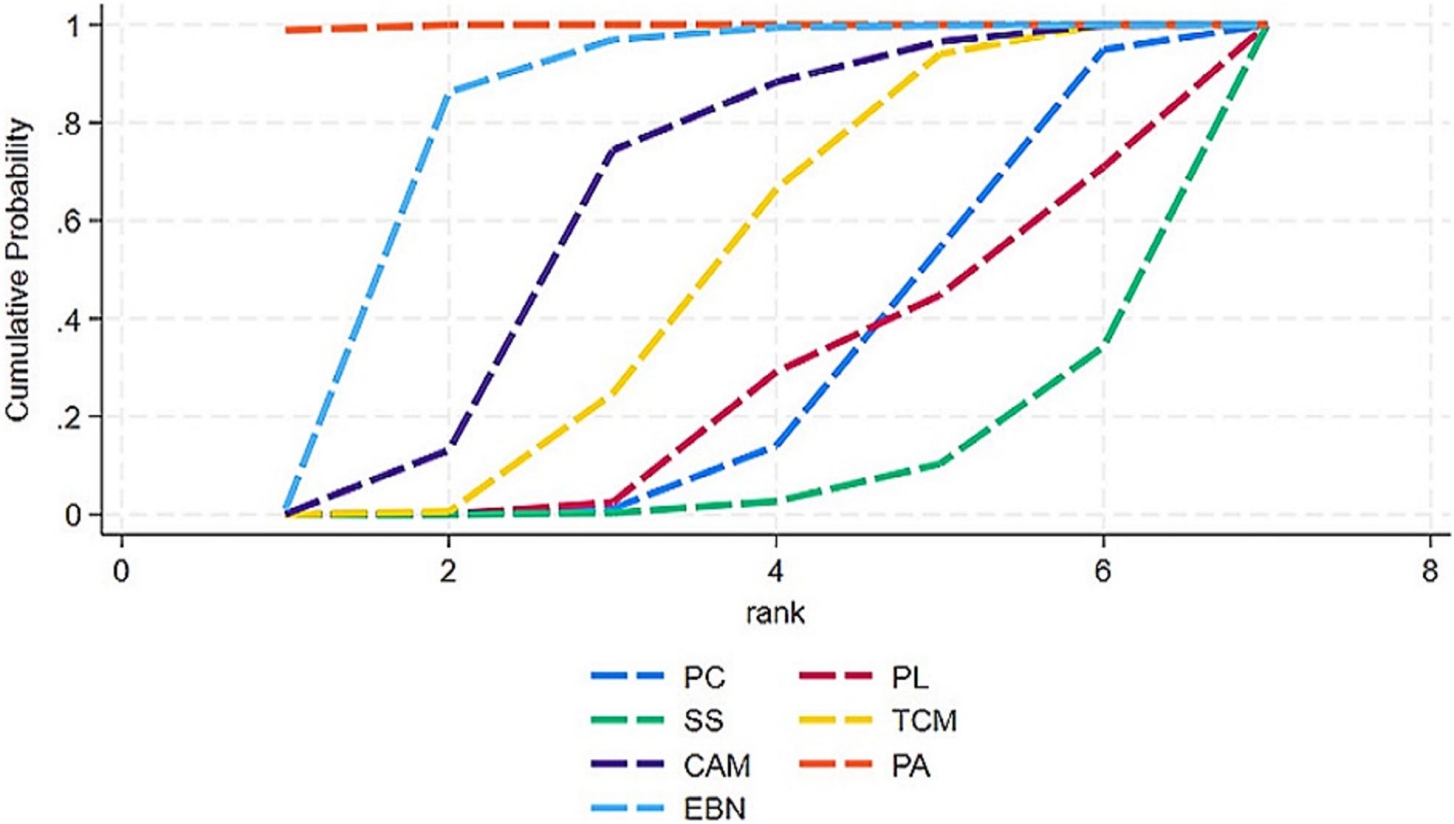
Integrated SUCRA diagram. Interventions with larger cumulative ranking areas demonstrate superior efficacy. CAM, Complementary and alternative medicine; EBN, evidence-based nursing; PAs, physical agents; PC, passive control; PL, placebo; SS, Sham stimulation; TCM, traditional Chinese medicine.

The primary results of the network meta-analyses are illustrated in Figure 4. Table 3 shows the comparative effectiveness of different treatments. Among these, PAs (SMDs = 2.06–2.32) and EBN (SMDs = 0.85–1.11) relieved constipation as compared to passive control, placebo, and sham stimulation. The contribution matrix of direct evidence is provided in Supplementary material 4. Treatment rankings based on cumulative probability plots and SUCRAs are shown in Figures 5, 6 and Table 4. The most effective intervention for relieving constipation was PAs (99.8%), followed by EBN (80.6%), CAM (62.1%), and TCM (47.6%).

**Table 3:** League table.

**Intervention**	**SS**	**PL**	**PC**	**TCM**	**CAM**	**EBN**	**PA**
**SS**							
**PL**	-0.22 (-1.03,0.59)						
**PC**	-0.26 (-0.68,0.17)	-0.04 (-0.72,0.65)					
**TCM**	**0.41 (0.07,0.75)**	0.19 (-0.54,0.93)	0.16 (-0.09,0.40)				
**CAM**	0.64 (-0.10,1.38)	**0.42 (0.11,0.73)**	0.39 (-0.22,1.00)	0.23 (-0.43,0.89)			
**EBN**	**1.11 (0.41,1.81)**	**0.89 (0.01,1.77)**	**0.85 (0.29,1.41)**	**0.70 (0.08,1.31)**	0.47 (-0.36,1.30)		
**PA**	**2.32 (1.55,3.09)**	**2.10 (0.98,3.22)**	**2.06 (1.19,2.94)**	**1.91 (1.07,2.75)**	**1.68 (0.61,2.75)**	**-1.21 (-2.25, -0.17)**	

Results are expressed as standardized mean difference and 95% confidence intervals. For each comparison (columns vs. rows), a difference >0 indicates that the intervention in the row outperforms the comparator in the columns. Bold numbers indicate statistically significant results. SS, Sham stimulation; PL, placebo; PC, passive control; TCM, traditional Chinese medicine; CAM, complementary and alternative medicine; EBN, evidence-based nursing; PAs, physical agents.

**Table 4:** Treatment ranking.

Rank	Treatment node	SUCRA	PrBest	Mean rank
1st	SS	7.9	0.0	6.5
2nd	PL	24.6	0.0	5.5
3rd	PC	27.4	0.0	5.4
4th	TCM	47.6	0.0	4.1
5th	CAM	62.1	0.1	3.3
6th	EBN	80.6	1	2.2
7th	PA	99.8	98.8	1

SS, Sham stimulation; PL, placebo; PC, passive control; TCM, traditional Chinese medicine; CAM, complementary and alternative medicine; EBN, evidence-based nursing; PAs, physical agents.

### 3.7 Heterogeneity analyses and publication bias

There was moderate heterogeneity (I^2^ = 72.6%, *p* < 0.001) among the included studies (Supplementary material 5.1). While subgroup analyses and meta-regression (Supplementary material 5.2) failed to identify clear moderators, sequential exclusion of individual studies demonstrated that Huang et al.’s trial (50) contributed disproportionately to heterogeneity (Supplementary material 5.3). Sensitivity analyses confirmed the robustness of our primary findings, with effect estimates remaining stable across model specifications (Supplementary material 5.4). Funnel plot analysis and Egger’s test did not indicate significant evidence of publication bias (Supplementary material 5.5).

### 3.8 Grading of evidence

Using the GRADE framework, we found considerable variability in evidence quality across outcomes (Supplementary material 6). The majority of comparisons (66.7%) were rated as low certainty, followed by very low (22.2%) and moderate (11.1%) certainty evidence. The evidence was downgraded primarily due to imprecision (wide 95% confidence intervals) and study limitations (inadequate blinding and small sample sizes).

## 4 Discussion

This is the first NMA to systematically quantify the comparative effectiveness of non-pharmacological interventions for constipation management in PD, synthesizing data from 12 randomized controlled trials encompassing 881 individuals. The analysis yielded a clear efficacy hierarchy among the four intervention categories examined: physical agent (PA) therapies demonstrated superior effectiveness, followed by evidence-based nursing (EBN), while complementary and alternative medicine (CAM) and traditional Chinese medicine (TCM) showed more modest effects. These findings align with and extend previous pairwise meta-analytic results, providing robust evidence that both PA and EBN interventions produce statistically and clinically significant improvements in PD with constipation. The consistency between direct and indirect comparisons strengthens confidence in these results, particularly for PA modalities, which presented the most pronounced treatment effects.

Physical agents, such as infrared radiation and repetitive transcranial magnetic stimulation, have gained widespread clinical acceptance for managing neuropathic pain, stroke, depression, and constipation, owing to their favorable tolerance, painlessness, fewer side effects, and convenience (50, 53). Huang et al. conducted high-frequency repeated magnetic stimulation on 48 patients with PD experiencing constipation and demonstrated that this modality may alleviate constipation by stimulating the nervous plexus surrounding the rectum (50), promoting blood circulation and movement of the rectum, and coordinating activation of defecation-related musculature (16). Additionally, physical stimulation may improve intestinal excretion by enhancing the mean colonic transit time and harmonizing the pelvic floor in patients suffering from slow-transit constipation (54).

Several studies have indicated the potential benefits of physical therapies such as transcranial pulsed electromagnetic and repetitive transcranial magnetic stimulation in Parkinson’s disease (55–58). Recently, transcutaneous auricular vagus nerve stimulation, a physical stimulation method distinct from invasive vagus nerve stimulation, has also been used in Parkinson’s disease to ameliorate Parkinsonian gait disorders by stimulating the auricular branch of the vagus nerve (59, 60). However, these investigations primarily focused on the management of motor symptoms, leaving non-motor aspects such as constipation comparatively underexplored. More large-scale, high-quality RCTs are warranted in the future to validate the efficacy of various physical therapies for gastrointestinal dysfunction in PD populations and to explore optimal treatment protocols regarding dosage, parameters (frequency and duration), and treatment intervals, thereby facilitating the development of standardized PA regimens and promoting their clinical translation for PD-related constipation.

As a novel nursing model grounded in evidence-based practice, EBN explicitly and judiciously uses a theoretical basis alongside the latest research evidence and takes into account the specific situation and needs of individuals (61). This approach represents an inevitable trend within modern medicine and has great potential for enhancing the satisfaction of nursing care while improving the quality of life among patients affected by PD with constipation (62). Recent study by Geng et al (63). established a tailored EBN intervention scheme suitable for managing constipation in individuals with PD through a literature review and two rounds of expert consultation, and the application effect was ideal among elderly PD patients. Previous research has also shown that identifying and managing gastrointestinal symptoms through EBN management has the potential to improve PD patients’ bowel-specific and global health outcomes (64).In summary, EBN is a fluid process of change (65), and the current evidence for PD care is limited (66), which requires a continuous, deliberate, and systematic evaluation to achieve better outcomes for PD with constipation.

Several limitations should be acknowledged when interpreting our NMA findings. (1) The paucity of included studies, particularly the single trial evaluating PAs. Combined with its methodological shortcomings (notably lack of blinding), variability in treatment effects, and limited follow-up duration, necessitates cautious interpretation of PA’s designation as the optimal non-pharmacological intervention for PD with constipation; (2) The relatively small sample sizes, both overall and under each intervention classification, are likely to interfere with the stability and accuracy of the results; (3) High risk of bias. Although the TCM intervention type is currently the most applied non-pharmacological therapy, our assessment revealed that 66.7% of included TCM studies raised some concerns regarding bias risk, while 16.7% were classified as high risk, primarily due to inadequate randomization and blinding procedures. Furthermore, given the limitations of language (English and Chinese) and region (China, Malaysia, and Iran), there is a high likelihood of interfering with the reporting of treatment efficacy rankings. These observations underscore the need for future large-scale, well-designed, and culturally diverse RCTs to eliminate the effects of study quality, regional limitations, and ethnographic differences. (4) Moderate between-study heterogeneity (I^2^ = 72.6%) was identified, with case-by-case literature exclusion demonstrating the study by Huang et al. as a potential major contributor. This heterogeneity may not only inflate PA’s superiority but also potentially limit the generalizability of the findings. Given that 88.89% of outcomes were rated as very low or low certainty evidence in our GRADE assessment, future trials must prioritize larger sample sizes, more rigorous randomization and blinding protocols, and objective outcome reporting to establish reliable evidence for clinical practice.

## 5 Conclusion

Through a comprehensive network meta-analysis, we have evaluated the comparative efficacy of four distinct non-pharmacological interventions for PD with constipation. The current evidence suggests that PAs and EBN demonstrate clinically meaningful benefits, while TCM and CAM show relatively modest effects. However, these conclusions should be interpreted with caution due to limitations in study quantity and methodological quality. These findings underscore the imperative for more rigorous, large-scale randomized controlled trials using standardized protocols to establish robust evidence for optimizing therapeutic approaches.

## Data Availability

The original contributions presented in the study are included in the article/Supplementary material, further inquiries can be directed to the corresponding author.

## Footnotes

^1^ https://www.gradepro.org
