# Supplementary material for: Comparative efficacy of non-pharmacological interventions for Parkinson’s disease with constipation: a systematic review and network meta-analysis
Source: Front Neurol. 2025 Jun 2;16:1579556. doi: 10.3389/fneur.2025.1579556 (PMC12171382; doi:10.3389/fneur.2025.1579556)
Supplement: Supplementary file 1 [file Data_Sheet_1.pdf]

Supplementary Files

Index of supplementary materials

Supplementary 1. PRISMA checklist
Supplementary 2. Search strategy
Supplementary 3. Pairwise meta-analyses
Supplementary 4. The contribution matrix of direct evidence
Supplementary 5. Heterogeneity analyses and publication bias
    5.1 Heterogeneity analyses
    5.2 Subgroup analyses and meta-regression analyses
    5.3 Case-by-case literature exclusion
    5.4 Sensitivity analyses
    5.5 Publication bias
Supplementary 6. GRADE

Supplementary 1. PRISMA checklist

Section/Topic	Item #	Checklist Item	Reported on Page #
**TITLE**			
Title	1	Identify the report as a systematic review *incorporating a network meta-analysis (or related form of meta-analysis).*	1
**ABSTRACT**			
Structured summary	2	Provide a structured summary including, as applicable:<br>**Background:** main objectives<br>**Methods:** data sources; study eligibility criteria, participants, and interventions; study appraisal; and *synthesis methods, such as network meta-analysis.*<br>**Results:** number of studies and participants identified; summary estimates with corresponding confidence/credible intervals; *treatment rankings may also be discussed. Authors may choose to summarize pairwise comparisons against a chosen treatment included in their analyses for brevity.*<br>**Discussion/Conclusions:** limitations; conclusions and implications of findings.<br>**Other:** primary source of funding; systematic review registration number with registry name.	1
**INTRODUCTION**			
Rationale	3	Describe the rationale for the review in the context of what is already known, *including mention of why a network meta-analysis has been conducted.*_	2
Objectives	4	Provide an explicit statement of questions being addressed, with reference to participants, interventions, comparisons, outcomes, and study design (PICOS).	2
**METHODS**			
Protocol and registration	5	Indicate whether a review protocol exists and if and where it can be accessed (e.g., Web address);	2

		and, if available, provide registration information, including registration number.	
Eligibility criteria	6	Specify study characteristics (e.g., PICOS, length of follow-up) and report characteristics (e.g., years considered, language, publication status) used as criteria for eligibility, giving rationale. *Clearly describe eligible treatments included in the treatment network, and note whether any have been clustered or merged into the same node (with justification).*	3; Table 1
Information sources	7	Describe all information sources (e.g., databases with dates of coverage, contact with study authors to identify additional studies) in the search and date last searched.	2; Supplementary 2
Search	8	Present full electronic search strategy for at least one database, including any limits used, such that it could be repeated.	2; Supplementary 2
Study selection	9	State the process for selecting studies (i.e., screening, eligibility, included in systematic review, and, if applicable, included in the meta-analysis).	3-4; Figure 1
Data collection process	10	Describe method of data extraction from reports (e.g., piloted forms, independently, in duplicate) and any processes for obtaining and confirming data from investigators.	3-4
Data items	11	List and define all variables for which data were sought (e.g., PICOS, funding sources) and any assumptions and simplifications made.	3-4
**Geometry of the network**	**S1**	Describe methods used to explore the geometry of the treatment network under study and potential biases related to it. This should include how the evidence base has been graphically summarized for presentation, and what characteristics were compiled and used to describe the evidence base to readers.	4-5
Risk of bias within individual studies	12	Describe methods used for assessing risk of bias of individual studies (including specification of whether this was done at the study or outcome level), and how this information is to be used in any data synthesis.	4; Figure 2
Summary measures	13	State the principal summary measures (e.g., risk ratio, difference in means). *Also describe the use of additional summary measures assessed, such as treatment rankings and surface under the*	4

		cumulative ranking curve (SUCRA) values, as well as modified approaches used to present summary findings from meta-analyses.	
Planned methods of analysis	14	Describe the methods of handling data and combining results of studies for each network meta-analysis. This should include, but not be limited to:<br>• *Handling of multi-arm trials;*<br>• *Selection of variance structure;*<br>• *Selection of prior distributions in Bayesian analyses; and*<br>• *Assessment of model fit.*	4
**Assessment of Inconsistency**	**S2**	Describe the statistical methods used to evaluate the agreement of direct and indirect evidence in the treatment network(s) studied. Describe efforts taken to address its presence when found.	4
Risk of bias across studies	15	Specify any assessment of risk of bias that may affect the cumulative evidence (e.g., publication bias, selective reporting within studies).	4-5
Additional analyses	16	Describe methods of additional analyses if done, indicating which were pre-specified. This may include, but not be limited to, the following:<br>• Sensitivity or subgroup analyses;<br>• Meta-regression analyses;<br>• *Alternative formulations of the treatment network; and*<br>• *Use of alternative prior distributions for Bayesian analyses (if applicable).*_	4-5
**RESULTS†**			
Study selection	17	Give numbers of studies screened, assessed for eligibility, and included in the review, with reasons for exclusions at each stage, ideally with a flow diagram.	5; Figure 1
**Presentation of network structure**	**S3**	Provide a network graph of the included studies to enable visualization of the geometry of the treatment network.	5-6; Figure 3
**Summary of network geometry**	**S4**	Provide a brief overview of characteristics of the treatment network. This may include commentary on the abundance of trials and randomized patients for the different interventions and pairwise comparisons in the network, gaps of evidence in the treatment network, and potential	5-6

		biases reflected by the network structure.	
Study characteristics	18	For each study, present characteristics for which data were extracted (e.g., study size, PICOS, follow-up period) and provide the citations.	5; Table 1
Risk of bias within studies	19	Present data on risk of bias of each study and, if available, any outcome level assessment.	5; Figure 2
Results of individual studies	20	For all outcomes considered (benefits or harms), present, for each study: 1) simple summary data for each intervention group, and 2) effect estimates and confidence intervals. *Modified approaches may be needed to deal with information from larger networks.*	5-6; Table 2; Supplementary 3 and 4
Synthesis of results	21	Present results of each meta-analysis done, including confidence/credible intervals. *In larger networks, authors may focus on comparisons versus a particular comparator (e.g. placebo or standard care), with full findings presented in an appendix. League tables and forest plots may be considered to summarize pairwise comparisons.* If additional summary measures were explored (such as treatment rankings), these should also be presented.	5; Supplementary 3
**Exploration for inconsistency**	**S5**	Describe results from investigations of inconsistency. This may include such information as measures of model fit to compare consistency and inconsistency models, *P* values from statistical tests, or summary of inconsistency estimates from different parts of the treatment network.	4
Risk of bias across studies	22	Present results of any assessment of risk of bias across studies for the evidence base being studied.	6; Supplementary 5.5
Results of additional analyses	23	Give results of additional analyses, if done (e.g., sensitivity or subgroup analyses, meta-regression analyses, *alternative network geometries studied, alternative choice of prior distributions for Bayesian analyses,* and so forth).	6; Supplementary 5 and 6
**DISCUSSION**			
Summary of evidence	24	Summarize the main findings, including the strength of evidence for each main outcome; consider their relevance to key groups (e.g., healthcare providers, users, and policy-makers).	6-8

Limitations	25	Discuss limitations at study and outcome level (e.g., risk of bias), and at review level (e.g., incomplete retrieval of identified research, reporting bias). *Comment on the validity of the assumptions, such as transitivity and consistency. Comment on any concerns regarding network geometry (e.g., avoidance of certain comparisons).*	7-8
Conclusions	26	Provide a general interpretation of the results in the context of other evidence, and implications for future research.	8
**FUNDING**			
Funding	27	Describe sources of funding for the systematic review and other support (e.g., supply of data); role of funders for the systematic review. This should also include information regarding whether funding has been received from manufacturers of treatments in the network and/or whether some of the authors are content experts with professional conflicts of interest that could affect use of treatments in the network.	8

Notes: PICOS = population, intervention, comparators, outcomes, study design.

* Text in italics indicates wording specific to reporting of network meta-analyses that has been added to guidance from the PRISMA statement.

† Authors may wish to plan for use of appendices to present all relevant information in full detail for items in this section.

Supplementary 2. Search strategy

Database: CINAHL Complete via EBSCO
S1: (MH "Parkinson Disease")<br>S2: SU Parkinson Disease OR SU Idiopathic Parkinson's Disease OR SU Lewy Body Parkinson's Disease OR SU Parkinson's Disease, Idiopathic OR SU Parkinson's Disease, Lewy Body OR SU Paralysis Agitans OR SU Parkinson's Disease OR SU Idiopathic Parkinson Disease OR SU Lewy Body Parkinson Disease OR SU Primary Parkinsonism OR SU Parkinsonism, Primary OR SU Parkinson Disease, Idiopathic<br>S3: S1 OR S2<br>S4: (MH "Constipation")<br>S5: SU Constipation OR SU Constipation OR SU Colonic Inertia OR SU Dyschezia<br>S6: S4 OR S5<br>S7: (MH "Randomized Controlled Trials")<br>S8: SU Randomized Controlled Trials OR SU Randomized Controlled Trials as Topic OR SU Controlled Clinical Trials as Topic<br>S9: S7 OR S8<br>S10: SU randomi?ed OR SU randomi?action OR SU randomi?ing AND SU trial<br>S11: SU assign* OR SU allocat* AND SU random*<br>S12: RCT<br>S13: S12 OR S10 OR S11<br>S14: S9 OR S13<br>S15: SU Animal Experimentation OR SU animal experiment* OR SU animal stud* OR SU animal physical conditioning OR SU animal research<br>S16: S3 AND S6 AND S13<br>S17: NOT S15
**Database: Cochrane Library (CENTRAIL)**
#1: MeSH descriptor: [Parkinson Disease] explode all trees<br>#2: (Parkinson Disease):ti,ab,kw OR (idiopathic parkinson s disease):ti,ab,kw OR (lewy body parkinson s disease):ti,ab,kw OR (parkinson s disease idiopathic):ti,ab,kw OR (parkinson s disease lewy body):ti,ab,kw OR (paralysis agitans):ti,ab,kw OR (parkinson s disease):ti,ab,kw OR (idiopathic parkinson disease):ti,ab,kw OR (lewy body parkinson disease):ti,ab,kw OR (primary parkinsonism):ti,ab,kw OR (parkinsonism primary):ti,ab,kw OR (parkinson disease idiopathic):ti,ab,kw<br>#3: #1 OR #2<br>#4: MeSH descriptor: [Constipation] explode all trees<br>#5: (Constipation):ti,ab,kw OR (Constipation):ti,ab,kw OR (Colonic Inertia):ti,ab,kw OR (Dyschezia):ti,ab,kw<br>#6: #4 OR #5<br>#7: MeSH descriptor: [Randomized Controlled Trial] explode all trees<br>#8: MeSH descriptor: [Randomized Controlled Trials as Topic] explode all trees<br>#9: MeSH descriptor: [Controlled Clinical Trials as Topic] explode all trees<br>#10: #7 OR #8 OR #9<br>#11: ((randomi?ed or randomi?ation or randomi?ing) and trial) or RCT or (random* and

(assign* or allocat*))<br>#12: #10 OR #11<br>#13: MeSH descriptor:[Animal Experimentation] explode all trees<br>#14: animal experiment* or animal stud* or animal physical conditioning or animal research<br>#15: #13 OR #14<br>#16: #3 AND #6 AND #12<br>#17: #13 NOT #16
**Database: Embase**
#1: ‘parkinson disease’/exp<br>#2: ‘idiopathic parkinsonism’:ab,ti OR ‘lewy bodies of parkinson disease’:ab,ti OR ‘lewy bodies of parkinson`s disease’:ab,ti OR ‘lewy bodies of parkinsons disease’:ab,ti OR ‘lewy body parkinson`s disease’:ab,ti OR ‘lewy body parkinsons disease’:ab,ti OR ‘lewy body parkinson disease’:ab,ti OR ‘paralysis agitans’:ab,ti OR ‘parkinson dementia complex’:ab,ti OR ‘parkinson`s disease’:ab,ti OR ‘parkinsons disease’:ab,ti OR ‘primary parkinsonism’:ab,ti OR ‘parkinson disease’:ab,ti<br>#3: #1 OR #2<br>#4: ‘constipation’/exp<br>#5: obstipation:ab,ti OR ‘rectal constipation’:ab,ti OR ‘slow transit constipation’:ab,ti OR constipation:ab,ti<br>#6: #4 OR #5<br>#7: ‘randomized controlled trial (topic)’/exp<br>#8: ‘randomized controlled trial’/exp<br>#9: ‘controlled clinical trial’/exp<br>#10: #7 OR #8 OR #9<br>#11: ((‘randomi?ed’:ti,ab OR ‘randomi?ation’:ti,ab OR ‘randomi?ing’:ti,ab) AND ‘trial’:ti,ab OR ‘rct’:ti,ab OR (‘random*’:ti,ab AND (‘assign*’:ti,ab OR ‘allocat*’:ti,ab))<br>#12: #10 OR #11<br>#13: #3 AND #6 AND #12<br>#14: ‘animal experiment’/exp OR ‘animal experiment*’:ti,ab OR ‘animal stud*’:ti,ab OR ‘animal physical conditioning’:ti,ab OR ‘animal research’:ti,ab<br>#15: #13 NOT #14
**Database: PubMed**
(((“Parkinson Disease”[MeSH Terms] OR “Parkinson Disease”[Title/Abstract] OR “idiopathic parkinson s disease”[Title/Abstract] OR “lewy body parkinson s disease”[Title/Abstract] OR “parkinson s disease idiopathic”[Title/Abstract] OR “parkinson s disease lewy body”[Title/Abstract] OR “paralysis agitans”[Title/Abstract] OR “parkinson s disease”[Title/Abstract] OR “idiopathic parkinson disease”[Title/Abstract] OR “lewy body parkinson disease”[Title/Abstract] OR “primary parkinsonism”[Title/Abstract] OR “parkinsonism primary”[Title/Abstract] OR “parkinson disease idiopathic”[Title/Abstract]) AND (“Constipation”[MeSH Terms] OR “Constipation”[Title/Abstract] OR “colonic inertia”[Title/Abstract] OR “Dyschezia”[Title/Abstract])) AND ((“Randomized Controlled Trial”[Publication

Type] OR "Randomized Controlled Trials as Topic"[MeSH Terms] OR "Controlled Clinical Trials as Topic"[MeSH Terms]) OR ((((((randomi?ed) OR (randomi?action)) OR (randomi?ing)) AND (trial)) OR (RCT[Title/Abstract])) OR (((assign*[Title/Abstract]) OR (allocat*[Title/Abstract])) AND (random*[Title/Abstract]))))) NOT ("Animal Experimentation"[MeSH Terms] OR "animal experiment*"[Title/Abstract] OR "animal stud*"[Title/Abstract] OR "animal physical conditioning"[Title/Abstract] OR "animal research"[Title/Abstract])
**Database: Web of Science**
(TS=(Parkinson Disease) OR TS=(Parkinson Disease OR idiopathic parkinson s disease OR lewy body parkinson s disease OR parkinson s disease idiopathic OR parkinson s disease lewy body OR paralysis agitans OR parkinson s disease OR idiopathic parkinson disease OR lewy body parkinson disease OR primary parkinsonism OR parkinsonism primary OR parkinson disease idiopathic)) AND (TS=(Constipation) OR TS=(Constipation OR colonic inertia OR Dyschezia)) AND (TS=(Randomized Controlled Trial OR Randomized Controlled Trials as Topic OR Controlled Clinical Trials as Topic) OR TS=((randomi?ed OR randomi?action OR randomi?ing) AND trial) OR TS=(RCT) OR TS=(random* and (assign* OR allocat*))) NOT (TS=(animal experiment* OR animal stud* OR animal physical conditioning OR animal research))
**Database: Chinese National Knowledge Infrastructure (CNKI)**
SU%=('Parkinson's disease' + 'parkinson disease' + 'parkinsonian' + 'parkinson's disease' + 'parkinson syndrome' + 'parkinson's syndrome' + 'parkinsonism' + 'parkinson's syndrome' + 'Shaking palsy' + 'parkinson syndrome' + 'parkinson's disease' + 'parkinson') AND SU%=('Constipation' + 'astriction' + 'constipation' + 'severe constipation' + 'weak constipation' + 'Dry stool')
**Database: Wanfang databases**
Subject:("Parkinson's disease" OR "parkinson disease" OR "parkinsonian" OR "parkinson's disease" OR "parkinson syndrome" OR "parkinson's syndrome" OR "parkinsonism" OR "parkinson's syndrome" OR "Shaking palsy" OR "parkinson syndrome" OR "parkinson's disease" OR "parkinson") and Subject:("Constipation" OR "astriction" OR "constipation" OR "severe constipation" OR "weak constipation" OR "Dry stool")

Supplementary 3. Pairwise meta-analyses

Table S3 Pairwise meta-analyses

treatment1	treatment2	N of studies	SMD(95% CI)	*P* value	I^2 (%)
PC	TCM	4	-0.14 (-0.40, 0.11)	0.244	28
PL	CAM	3	-0.44 (-0.71, -0.17)	0.422	0
PC	CAM	1	-0.39 (-0.98, 0.19)	0.01	0
SS	TCM	2	-0.42 (-0.73, -0.10)	0.984	0
PC	EBN	1	**-0.88 (-1.39, -0.33)**	**0.01**	0
SS	PA	1	**-2.36 (-3.10, -1.62)**	**0.01**	0

Notes: Results in bold indicate significance at $p < 0.05$.

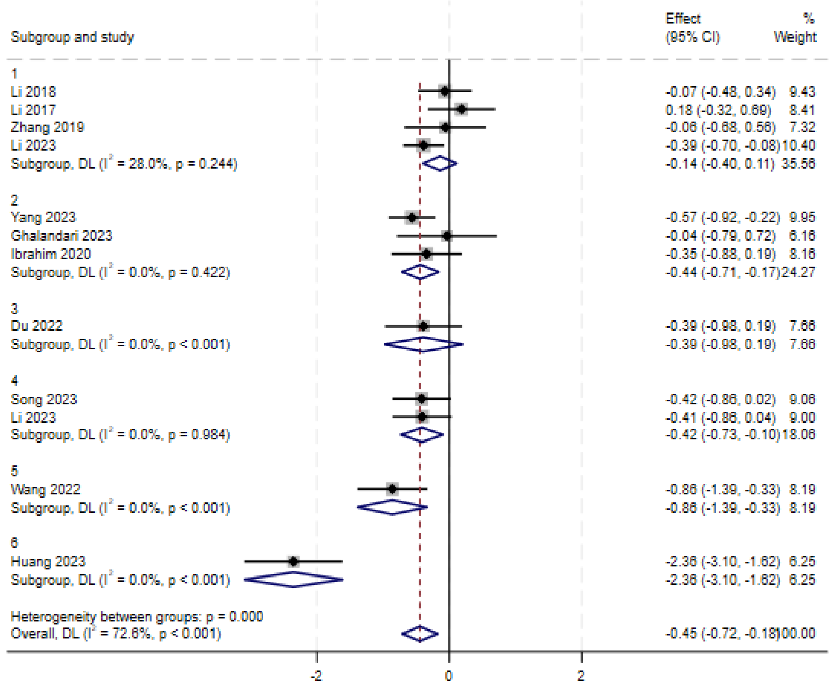

Figure S3 Forest of pairwise meta-analyses.

Supplementary 4. The contribution matrix of direct evidence

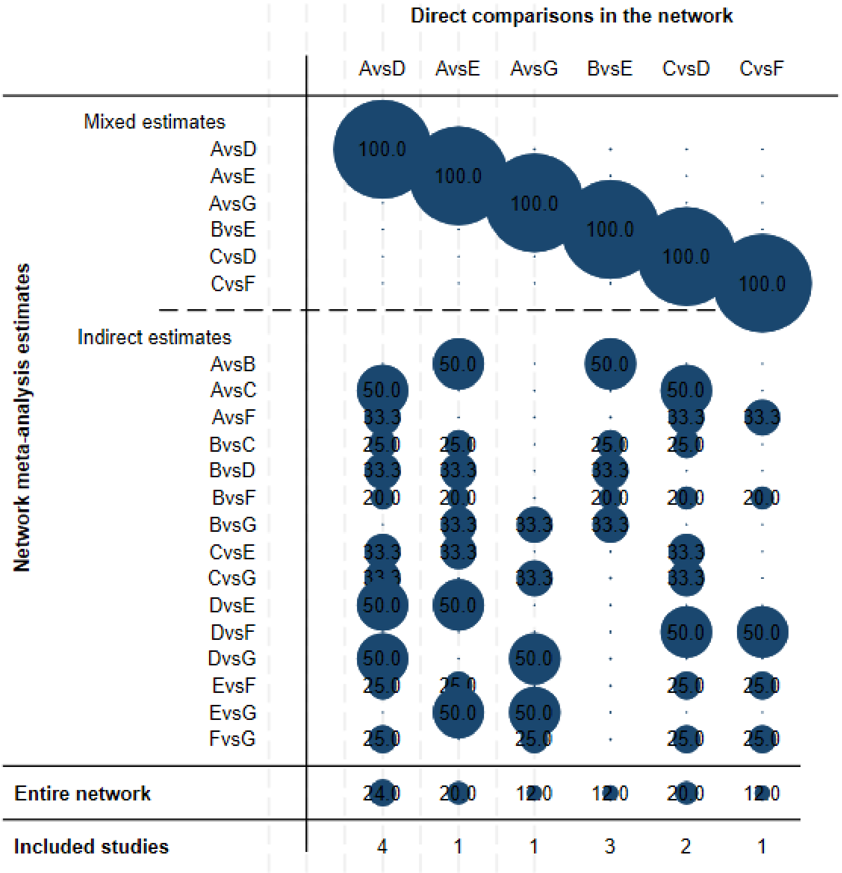

Figure S4 The contribution matrix of direct evidence. **Notes:** A = Passive control; B = Placebo; C = Sham stimulation; D = Traditional Chinese medicine; E = Complementary and alternative medicine; F = Physical agents; G = Evidence-based nursing.

Supplementary 5. Heterogeneity analyses and publication bias

5.1 Heterogeneity analyses

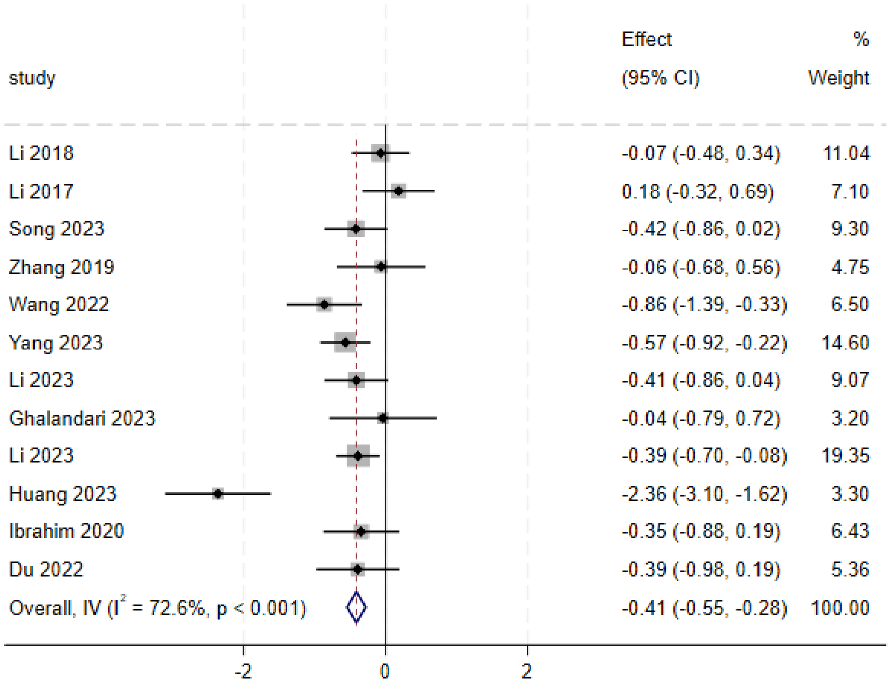

Figure S5.1 Forest of heterogeneity analyses.

5.2 Subgroup analyses and meta-regression analyses

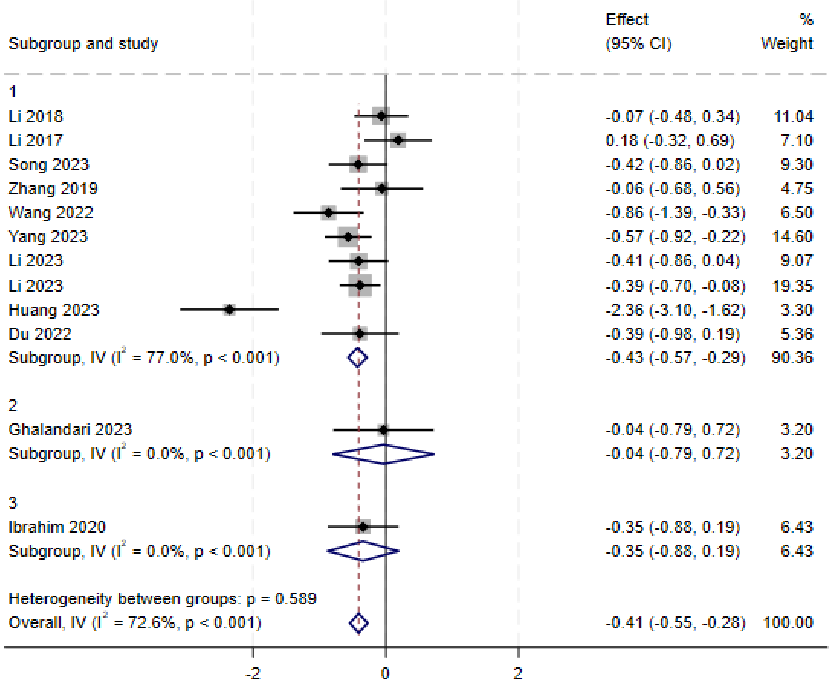

Figure S5.2a Subgroup analyses of region. **Notes:** subgroup 1, China; subgroup 2, Iran; subgroup 3, Malaysia.

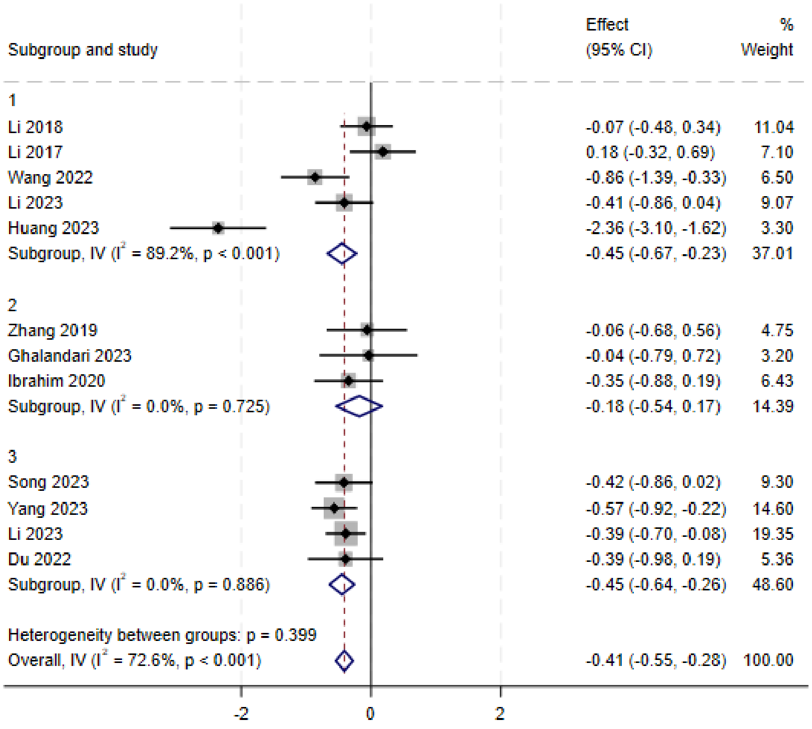

Figure S5.1b Subgroup analyses of follow-up time. **Notes:** subgroup 1, follow-up time ≤ 4 weeks; subgroup 2, 4 weeks < follow-up time < 8 weeks; subgroup 3, follow-up time ≥ 12 weeks.

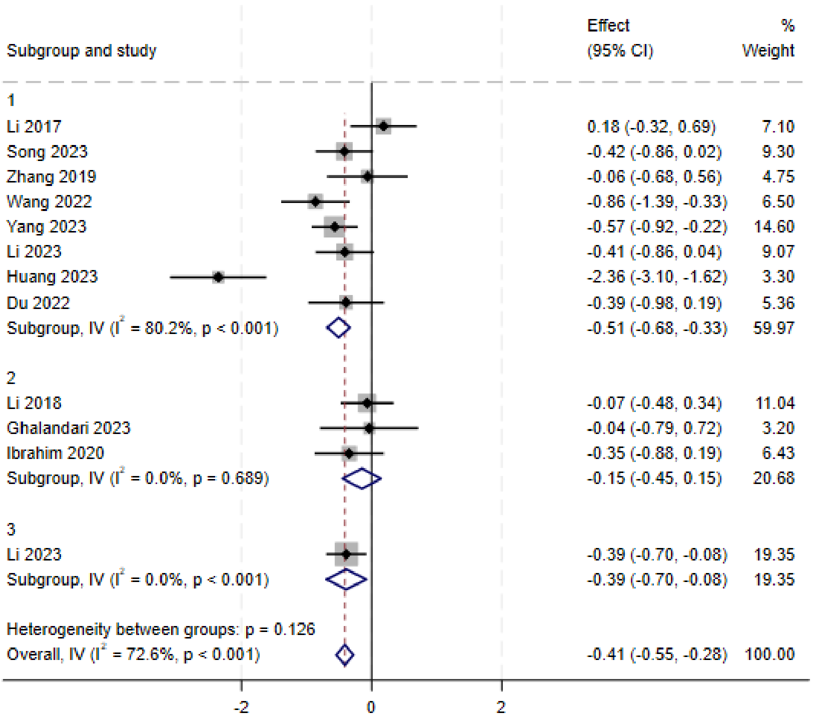

Figure S5.2c Subgroup analyses of outcome. **Notes:** subgroup 1, PAC-QOL; subgroup 2, UPDRS-III; subgroup 3, PDQ39.

Table S5.2 Meta-regression analyses

Subgroup	Coefficient	Std. err.	*t*	*P* > |*t*|	[95% conf. interval]	
_cons	1.966967	0.0689362	28.53	0.000	1.81524	2.118695

Note: $p > 0.05$ indicate no significance.

5.3 Case-by-case literature exclusion

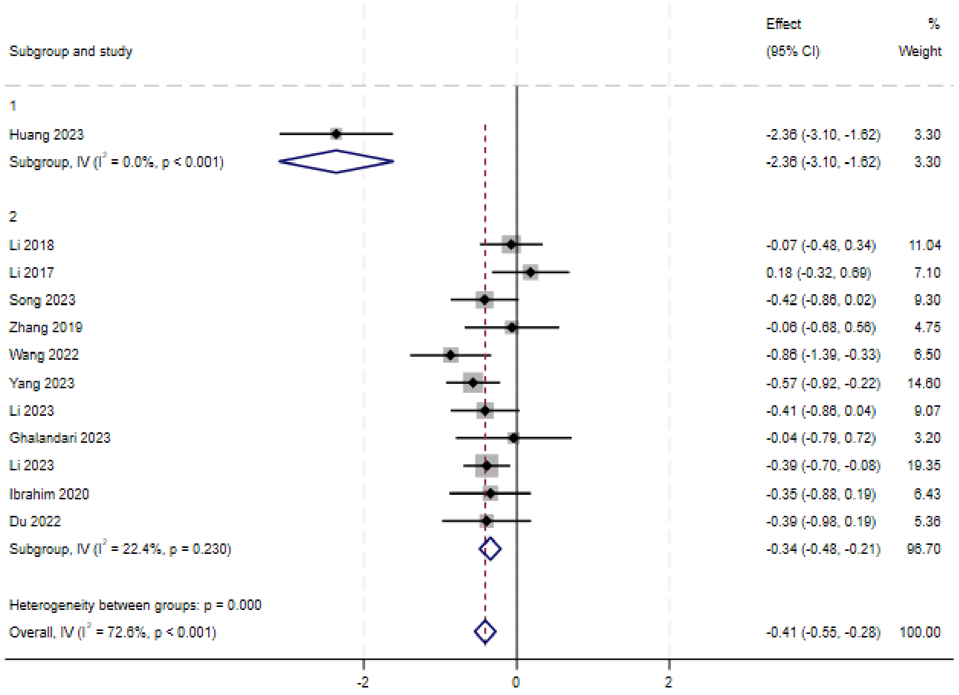

Figure S5.3 Forest of case-by-case literature exclusion.

5.4 Sensitivity analyses

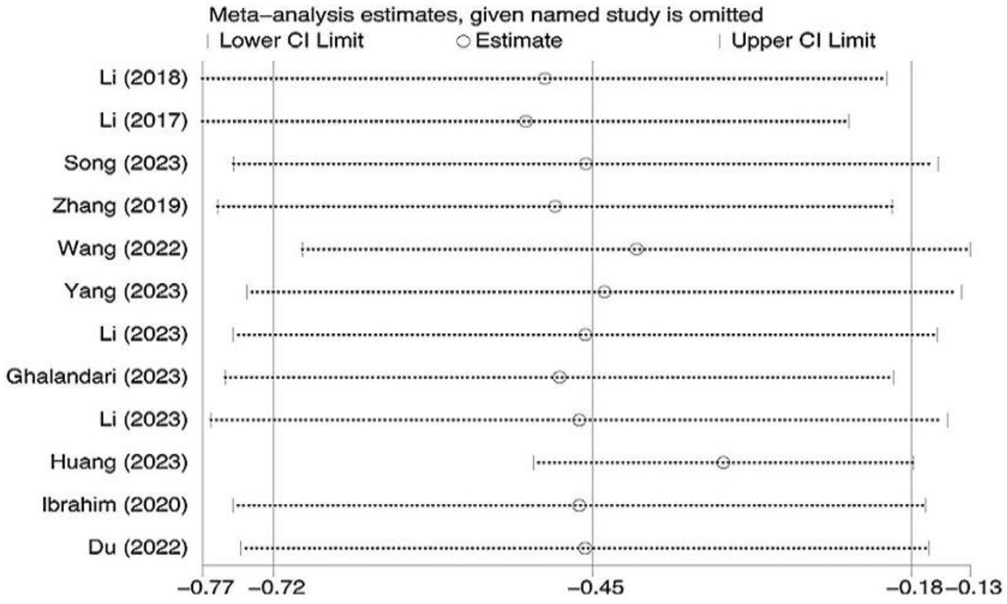

Figure S5.4 The sensitivity analyses.

5.5 Publication bias

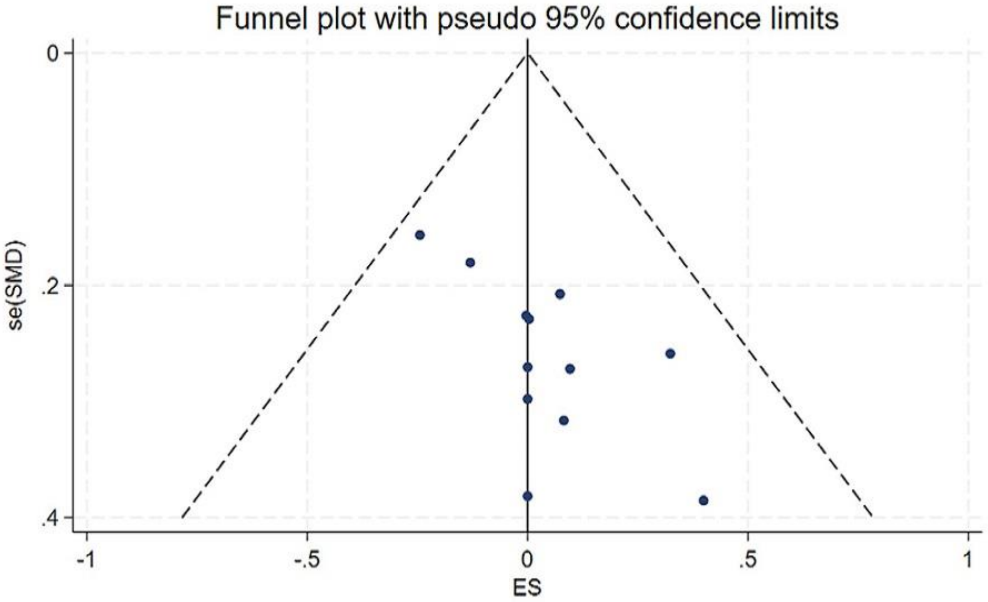

Figure S5.5 The results of funnel plot. **Notes:** ES, effect size; SMD, standardized mean difference.

Table S5.5 The result of Egger' s test.

Std_ Eff	Coefficient	Std. err.	t	*P* > |t|	[95% conf. interval]	
slope	-0.0474544	0.5189025	-0.09	0.929	-1.203641	1.108732
bias	-1.577746	2.172941	-0.73	0.484	-6.41936	3.263867

Note: $p > 0.05$ indicate no significant publication bias.

Supplementary 6. GRADE

Author(s): Zhang
Question: non-pharmacological intervention compared to control for Parkinson's disease with constipation
Setting: Importance - CRITICAL

Certainty assessment							№ of patients		Effect		Certainty	Importance
№ of studies	Study design	Risk of bias	Inconsistency	Indirectness	Imprecision	Other considerations	Experiment	Control	Relative (95% CI)	Absolute (95% CI)		
**TCM (assessed with: PAC-QOL)**												
4	randomised trials	serious [a]	not serious [b]	not serious [c]	very serious [d, e]	none	129	129	-	SMD **0.22 SD lower** (0.47 lower to 0.03 higher)	⊕◯◯◯ Very low [a, b, c, d, e]	CRITICAL
**CAM (assessed with: PAC-QOL)**												
2	randomised trials	not serious	not serious [b]	not serious [c]	very serious [d, e]	none	88	86	-	SMD **0.52 SD lower** (0.82 lower to 0.22 lower)	⊕⊕◯◯ Low [b, c, d, e]	CRITICAL
**EBN (assessed with: PAC-QOL)**												

1	randomised trials	not serious	not serious [b]	not serious [c]	serious [e]	none	30	30	-	SMD **0.86 SD lower** (1.39 lower to 0.33 lower)	⊕⊕⊕◯ Moderate [b, c, e]	CRITICAL
**PA (assessed with: PAC-QOL)**												
1	randomised trials	serious [a]	not serious [b]	not serious [c]	serious [e]	none	24	24	-	SMD **2.36 SD lower** (3.1 lower to 1.62 lower)	⊕⊕◯◯ Low [a, b, c, e]	CRITICAL

Notes: CI: confidence interval; SMD: standardised mean difference.

Explanations

a. 2/3 of included studies were biased in terms of randomization, allocation concealment or blinding.

b. If heterogeneity between studies is assessed as $50\% < I^2 < 75\%$, the assessment rating is reduced by one level; if $I^2 \geq 75\%$, it is reduced by two levels.

c. Degradation is generally not considered.

d. 95% confidence intervals crossed equivalence line.

e. Sample size < 400.

Author(s): Zhang
Question: non-pharmacological intervention compared to control for Parkinson's disease with constipation
Setting: Importance - IMPORTANT

Certainty assessment							№ of patients		Effect		Certainty	Importance
№ of studies	Study design	Risk of bias	Inconsistency	Indirectness	Imprecision	Other considerations	Experiment	Control	Relative (95% CI)	Absolute (95% CI)		
**TCM (assessed with: UPDRS-III)**												
1	randomised trials	serious [a]	not serious [b]	not serious [c]	very serious [d, e]	none	47	46	-	SMD **0.07 SD lower** (0.48 lower to 0.34 higher)	⊕◯◯◯ Very low [a, b, c, d, e]	IMPORTANT
**TCM (assessed with: PDQ39)**												
1	randomised trials	serious [a]	not serious [b]	not serious [c]	serious [e]	none	83	83	-	SMD **0.39 SD lower** (0.7 lower to 0.08 lower)	⊕⊕◯◯ Low [a, b, c, e]	IMPORTANT
**CAM (assessed with: UPDRS-III)**												
3	randomised trials	not serious	not serious [b]	not serious [c]	very serious [d, e]	none	106	104	-	SMD **0.44 SD lower** (0.71 lower to 0.17 lower)	⊕⊕◯◯ Low [b, c, d, e]	IMPORTANT

**CAM (assessed with: PDQ39)**												
2	randomised trials	not serious	not serious [b]	not serious [c]	very serious [d, e]	none	92	91	-	SMD **0.57 SD lower** (0.92 lower to 0.22 lower)	⊕⊕◯◯ Low [b, c, d, e]	IMPORTANT
**PA (assessed with: UPDRS-III)**												
1	randomised trials	serious [a]	not serious [b]	not serious [c]	serious [e]	none	24	24	-	SMD **2.36 SD lower** (3.1 lower to 1.62 lower)	⊕⊕◯◯ Low [a, b, c, e]	IMPORTANT

Notes: CI: confidence interval; SMD: standardised mean difference.

Explanations

a. 2/3 of included studies were biased in terms of randomization, allocation concealment or blinding.

b. If heterogeneity between studies is assessed as $50\% < I^2 < 75\%$, the assessment rating is reduced by one level; if $I^2 \geq 75\%$, it is reduced by two levels.

c. Degradation is generally not considered.

d. 95% confidence intervals crossed equivalence line.

e. Sample size < 400.

Table S6 GRADE Assessment

Intervention	TCM			CAM			EBN	PA	
**Outcome**	PAC-QOL	UPDRS-III	PDQ39	PAC-QOL	UPDRS-III	PDQ39	PAC-QOL	PAC-QOL	UPDRS-III
**number of studies**	4	1	1	2	3	2	1	1	1
**Sample size**	258	93	166	174	210	183	60	48	48
**Mean follow-up time (weeks)**	7.25	4	12	12	9.33	10	2	4	4
**Limitations**	-1	-1	-1	0	0	0	0	-1	-1
**Explanations**	2/3 of included studies were biased in terms of randomization, allocation concealment or blinding.	2/3 of included studies were biased in terms of randomization, allocation concealment or blinding	2/3 of included studies were biased in terms of randomization, allocation concealment or blinding	/	/	/	/	2/3 of included studies were biased in terms of randomization, allocation concealment or blinding	2/3 of included studies were biased in terms of randomization, allocation concealment or blinding
**Inconsistency**	0	0	0	0	0	0	0	0	0
**Explanations**	Mild heterogeneity, I2 = 27.7%.	I2 = 0.0%	I2 = 0.0%	I2 = 0.0%	I2 = 0.0%	I2 = 0.0%	I2 = 0.0%	I2 = 0.0%	I2 = 0.0%
**Indirectness**	0	0	0	0	0	0	0	0	0

**Explanations**	Degradation is generally not considered.	Degradation is generally not considered.	Degradation is generally not considered.	Degradation is generally not considered.	Degradation is generally not considered.	Degradation is generally not considered.	Degradation is generally not considered.	Degradation is generally not considered.	Degradation is generally not considered.
**Imprecision**	-2	-2	-1	-2	-2	-2	-1	-1	-1
**Obvious benefits or harms**	No	No	No	No	No	No	No	No	No
**Explanations**	Sample size < 400 and 95% confidence intervals across equivalence lines.	Sample size < 400 and 95% confidence intervals across equivalence lines.	Sample size < 400.	Sample size < 400 and 95% confidence intervals across equivalence lines.	Sample size < 400 and 95% confidence intervals across equivalence lines.	Sample size < 400 and 95% confidence intervals across equivalence lines.	Sample size < 400.	Sample size < 400.	Sample size < 400.
**publication bias**	0	0	0	0	0	0	0	0	0
**Importance**	8 - Critical	6 - important	5 - important	8 - Critical	6 - important	5 - important	8 - Critical	8 - Critical	6 - important
**Certainty**	very low	very low	Low	Low	Low	Low	Moderate	Low	Low
	D	D	C	C	C	C	B	C	C
